# Fine-tuning of Genome-Wide Polygenic Risk Scores and Prediction of Gestational Diabetes in South Asian Women

**DOI:** 10.1038/s41598-020-65360-y

**Published:** 2020-06-02

**Authors:** Amel Lamri, Shihong Mao, Dipika Desai, Milan Gupta, Guillaume Paré, Sonia S. Anand

**Affiliations:** 1 Department of Medicine, McMaster University Hamilton, Ontario, Canada; 2 Population Health Research Institute (PHRI), Hamilton, Ontario, Canada; 3 Canadian Collaborative Research Network (CCRN), Brampton, ON Canada; 4 Department of Pathology and Molecular Medicine, McMaster University, Hamilton, Ontario, Canada; 5 Department of Health Research Methods, Evidence, and Impact, McMaster University, Hamilton, Ontario, Canada

**Keywords:** Genome-wide association studies, Medical genomics, Genetic predisposition to disease, Gestational diabetes

## Abstract

Gestational diabetes Mellitus (GDM) affects 1 in 7 births and is associated with numerous adverse health outcomes for both mother and child. GDM is suspected to share a large common genetic background with type 2 diabetes (T2D). The aim of our study was to characterize different GDM polygenic risk scores (PRSs) and test their association with GDM using data from the South Asian Birth Cohort (START). PRSs were derived for 832 South Asian women from START using the pruning and thresholding (P + T), LDpred, and GraBLD methods. Weights were derived from a multi-ethnic and a white Caucasian study of the DIAGRAM consortium. GDM status was defined using South Asian-specific glucose values in response to an oral glucose tolerance test. Association with GDM was tested using logistic regression. Results were replicated in South Asian women from the UK Biobank (UKB) study. The top ranking P + T, LDpred and GraBLD PRSs were all based on DIAGRAM’s multi-ethnic study. The best PRS was highly associated with GDM in START (AUC = 0.62, OR = 1.60 [95% CI = 1.44–1.69]), and in South Asian women from UKB (AUC = 0.65, OR = 1.69 [95% CI = 1.28–2.24]). Our results highlight the importance of combining genome-wide genotypes and summary statistics from large multi-ethnic studies to optimize PRSs in South Asians.

## Introduction

Gestational diabetes mellitus (GDM) is defined as dysglycemia due to elevated blood glucose levels first identified during pregnancy, and is specifically defined based on glucose response to an oral glucose challenge test in pregnancy. GDM has been associated with numerous adverse health outcomes affecting mother and child, both during and after pregnancy^1,2^. Because of its increasing prevalence (~1 in 7 births), GDM has become a major health concern worldwide^3^. Nevertheless, the prevalence of GDM largely varies from one region of the globe to the other, and South Asian women have been shown to be at higher risk of GDM than white Caucasian women^3–7^.

Numerous genome-wide association studies (GWASs) and genome-wide association meta-analysis (GWAMAs) of glucose related traits and T2D have been conducted in non-gravid populations, and summary statistics from large consortia (e.g., MAGIC and DIAGRAM) are publicly available^8–17^. For instance, results from a DIAGRAM study lead by Mahajan *et al*., and which combines data for 26,488 T2D cases 83,964 controls from four different ethnic groups (Europeans, South Asians, East Asians and Mexicans) are available online. Summary statistics of DIAGRAM’s more recent GWAMAs (e.g. Scott *et al*.^10^: 26,676 T2D cases and 132,532 controls of European ancestry) were also released. By contrast, few studies of genetic determinants of GDM have been conducted or published. For instance, only three studies sought to identify genes associated with dysglycemia, GDM, and diabetes during pregnancy by GWAS^18–20^. Top signals from these studies were located within/near *CDKAL1*, *MTNR1B, GCKR*, *PCSK1, PPP1R3B* and *G6PC2*, which were previously known for their association with glucose metabolism and T2D^18,19^. In addition, other T2D associated loci (e.g., *TCF7L2, PPARG, CDKN2A/B, KCNQ1, GCK*, etc.) were also significantly associated with GDM when tested separately^21–45^, or combined in genetic risk scores (GRSs)^38,39,46–48^.

GRSs are used to capture genetic information at one or more loci. Most of published studies interested in complex traits/diseases and using GRSs typically combine data for a small number of single nucleotide polymorphisms (SNPs), and the predictive power of these GRSs is sub-optimal^49^. However, with the increased availability of genome-wide genotypes and publicly available data from large consortia, GRSs with a larger number of variants are being used, and the predictive value of these genome-wide polygenic risk scores (PRSs) has substantially improved^50,51^.

PRSs can be derived using different approaches, however, these require both summary statistics from an external GWAS, and genetic data from a reference panel for between-variants linkage disequilibrium LD (LD) calculations. Pruning and thresholding (P + T) is a commonly used heuristic approach to derive PRSs in which variants are filtered based on an empirically determined P-value threshold. Linked variants are further clustered in different groups and SNPs with the highest significance (lowest P values) in each group are prioritized and included in the PRS, while variants of less significance within the group are pruned out^52^. Other programs have been shown to improve the predictive value of the scores by allowing the inclusion of a larger number of independent as well as linked variants into the score using different approaches. For instance, LDpred, another commonly used method, estimates the mean weight of each variant, assuming a prior knowledge of the genetic architecture of the trait (fraction causal), and using a Bayesian approach^53^. More recently, we developed the gradient boosted and LD adjusted (GraBLD) method, a new PRS building approach which applies principles of machine-learning to estimate SNP weights (gradient boosted regression trees), and regional LD adjustment^54^.

The following analysis was conducted in women participating in the South Asian Birth Cohort (START). The GDM case/control status of participants was ascertained using the South Asian-specific cut-offs established by Farrar *et al*. (fasting plasma glucose levels ≥5.2 mmol/L and/or 2-hour post load levels ≥7.2 mmol/L for cases)^4^, and self-reported GDM status was used if these measures were unavailable. The main objectives of this study are: 1) To compare the different methods and fine tune various parameters in order to characterize and derive the best PRS in START; 2) To investigate the association of the best PRS with GDM; and 3) To validate these results in South Asian women from UK Biobank^55^.

## Results

### Population characteristics

Table 1 shows the characteristics of South Asian women from START and UK Biobank included in the main and replication analysis respectively. Because of major differences in recruitment strategies, inclusion criteria and study protocols, South Asian women from the UK Biobank were of older age, and higher weight and body mass index (BMI) compared to START participants. Furthermore, the proportion of participants with GDM was significantly lower in the UK Biobank sample, as this was based on self-report, as opposed to results of an oral glucose tolerance test in START.

**Table 1:** Characteristics of women participants from the START and UK Biobank studies with available GDM status and genotype data.

	South Asian Women	
	START	UK Biobank
Number of Participants with GDM data	832	2,386
GDM, n (%)	301 (36.2%)	52 (2.2%)
Age, years	30.2 (4.0)	53.0 (8.1)^‡^
Height, cm	162.3 (6.2)^¥^	156.8 (5.9)^‡^
Weight, kg	62.6 (12.0)^¥^	67.7 (12.5)^‡^
BMI, kg/m^2^	23.8 (4.4)	27.5 (4.9)^‡^
Family history of diabetes, n (%)	334 (40.2)	1,556 (49.1)

Data are mean (standard deviation) unless otherwise indicated.^¥^ Pre-pregnancy values.^‡^ Values from baseline data. Abbreviations: BMI, Body mass index; GDM, Gestational diabetes; START, South Asian birth cohort.

### Characteristics of the best PRSs

In order to derive the optimal PRS, we compared results for: (1) two different sources of summary statistics (namely Mahajan *et al*., 2014^9^ *vs*. Scott *et al*., 2017^10^); (2) five different minimal sample size thresholds; (3) two templates for LD calculations; (4) three methods to derive the PRSs, and; (5) different P-value thresholds to filter out variants. Supplementary Fig. 1 illustrates the different tuning parameters used. All PRSs were ranked based on their area under the curve (AUC) from association tests with GDM, and the PRS with the highest AUC was designated as our top PRS.

#### Mahajan *vs*. scott based PRSs

**Figure 1:**
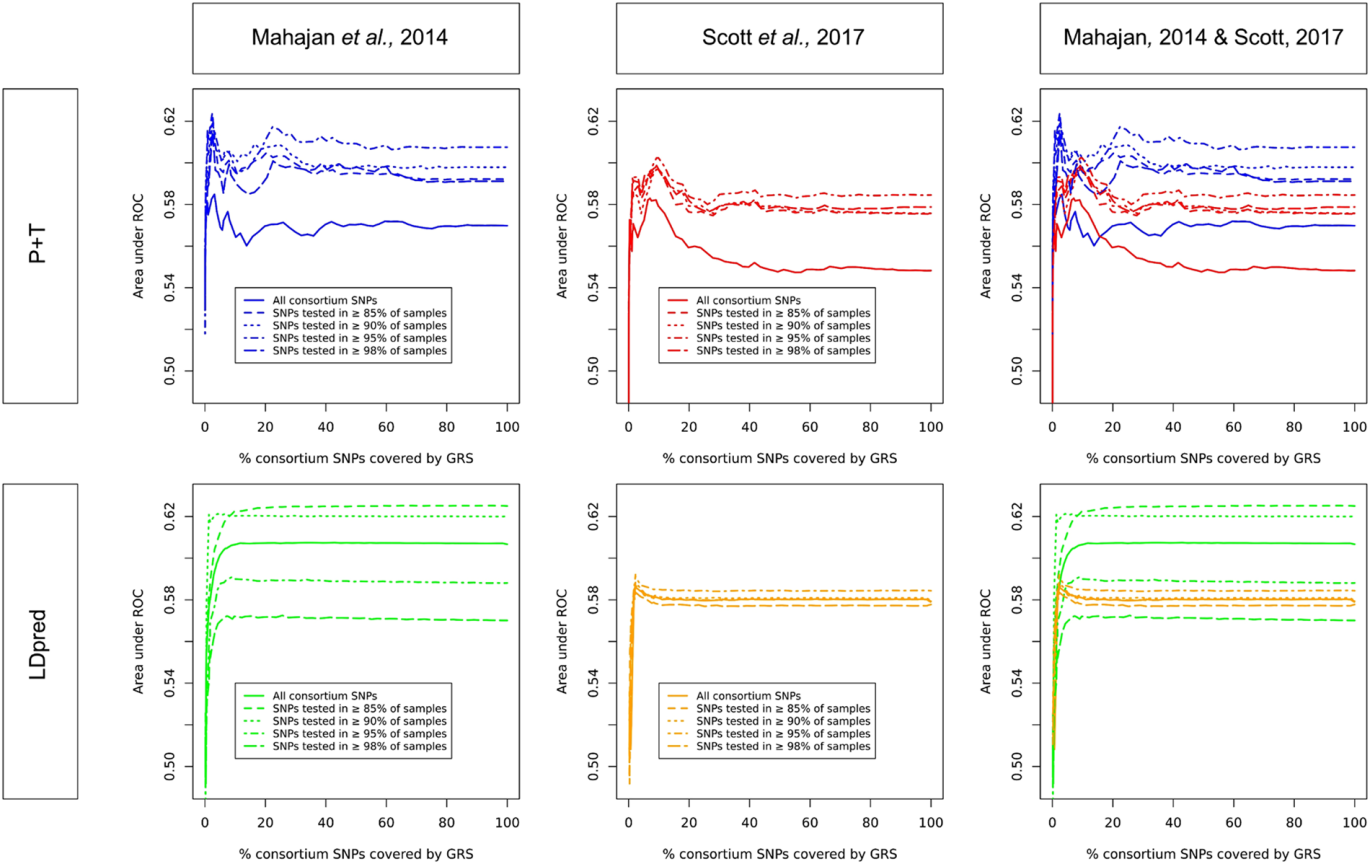
AUCs of the different P + T and LDpred PRSs based on Mahajan *et al*. and Scott *et al*. in South Asian women from START. Results from association tests with GDM, LD from 1000 Genomes. Abbreviations: AUC, Area under the curve; PRS, Polygenic risk score; P + T, Pruning and thresholding; SNP, Single nucleotide polymorphism; START, South Asian birth cohort; ROC, Receiver operating characteristic.

Summary statistics were derived from DIAGRAM’s trans-ethnic (Mahajan *et al*., 2014^9^) and white Caucasian (Scott *et al*., 2017^10^) GWAMAs. In Mahajan *et al*., 2,915,011 SNPs were tested for association with T2D in a wide range of samples (minimum N_samples_ = 25, maximum N_samples_ = 110,452), while 12,056,346 SNPs were tested in 4,731 to 159,208 samples in Scott *et al*. (Supplementary Table 1). Given the important disparity in the number of participants tested for each SNP (Supplementary Table 1 and Supplementary Fig. 2), we derived PRSs for which all variants were kept, as well as PRSs for which the list of variants was restricted to those tested in a larger number of samples (≥85, 90, 95 and 98% of the maximum N_sample_ in the GWAMA). The number of SNPs used in these different PRSs are shown in Supplementary Table 1. Our results show that, overall, PRSs that only include SNPs tested in a large number of samples (between 85% and 95% of the maximum N_samples_ of their respective consortia) perform better than PRSs where all variants are kept (including those tested in a small number of samples. Figure 1 and Supplementary Table 2).

The predictive value of the best Mahajan-based PRSs was higher than that of their Scott-based counterparts, independently of the method used (Fig. 1, Table 2, Supplementary Table 3).

**Table 2:** GDM association results of the best P + T, LDpred and GraBLD PRSs in South Asian women from the START and UK Biobank.

Method	Consortium	South Asian Women							
		START				UK Biobank			
		Beta	SE	P-value	AUC	Beta	SE	P-value	AUC
P + T	Mahajan *et al*., 2014	0.445	0.08	8.7 × 10^−9^	0.62	0.423	0.14	0.003	0.61
	Scott *et al*., 2017	0.370	0.07	7.86 × 10^−7^	0.60	0.280	0.14	0.05	0.57
GraBLD	Mahajan *et al.*, 2014	0.465	0.08	1.8 × 10^−9^	0.62	0.520	0.14	0.0003	0.64
	Scott *et al.*, 2017	0.317	0.07	1.61 × 10^−5^	0.59	0.388	0.14	0.006	0.61
LDpred	Mahajan *et al.*, 2014	0.461	0.07	2.18 × 10^−9^	0.62	0.527	0.14	0.0002	0.65
	Scott *et al.*, 2017	0.347	0.07	4.05 × 10^−6^	0.59	0.382	0.14	0.006	0.61

Results are from univariate association tests with GDM (LD from 1000 Genomes). Abbreviations: AUC, Area under the curve; GraBLD, Gradient boosted and LD adjusted; NA, Non applicable; P + T, pruning and thresholding; PRS, Polygenic risk score; SE, Standard error; START, South Asian Birth Cohort.

#### Impact of LD source

**Figure 2:**
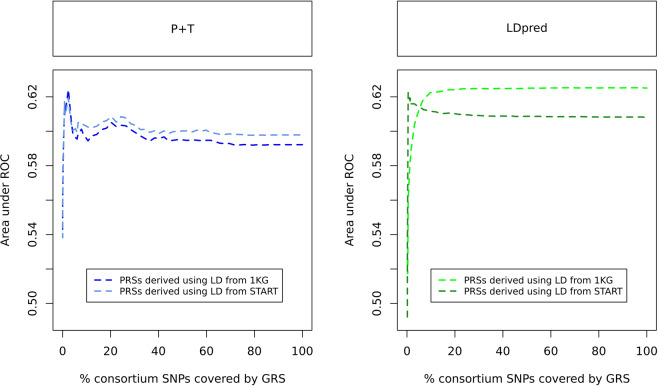
AUCs of the PRSs derived using LD from START and 1000 Genomes. Results are for Mahajan-based PRSs derived using SNPs tested in ≥85% of the study’s maximum N_samples_. Abbreviations: 1KG, 1000 Genomes; AUC, Area under the curve; PRS, Polygenic risk score; LD, Linkage disequilibrium; P + T, Pruning and thresholding; START, South Asian birth cohort; ROC, Receiver operating characteristic.

Since all three methods tested took into account between-variants LD, we used genotyping data from: 1) 1000 Genomes and 2) START studies as templates to estimate pairwise LDs and derive our PRSs (Fig. 1, Supplementary Fig. 3). Our results show that among the top rankig scores, the PRSs for which the LD was estimated using the 1000 Genomes mostly ranked higher than their START counterparts, independently of the method used, although this difference was substantially non-significant (Fig. 2, Supplemetary Table 3).

#### Effect of P-value thresholds

For each consortium study, LD source, and minimum N_sample_ tested, 64 different P-values (ranging from 5 × 10^−8^ to 1) were used as thresholds to filter out consortium variants to be included in the P + T and LDpred PRSs. Our results show that the inclusion of T2D associated variants with P-values higher than the usual 5 × 10^−8^ GWAS significance threshold in the PRS (i.e., less significant variants) always resulted in a considerable increase in AUC. Optimal AUCs were mostly reached for P-values > 0.01 for both Mahajan- and Scott-based PRSs (Fig. 1, Supplementary Table 2).

#### P + T vs. GraBLD vs. LDpred PRSs

**Figure 3:**
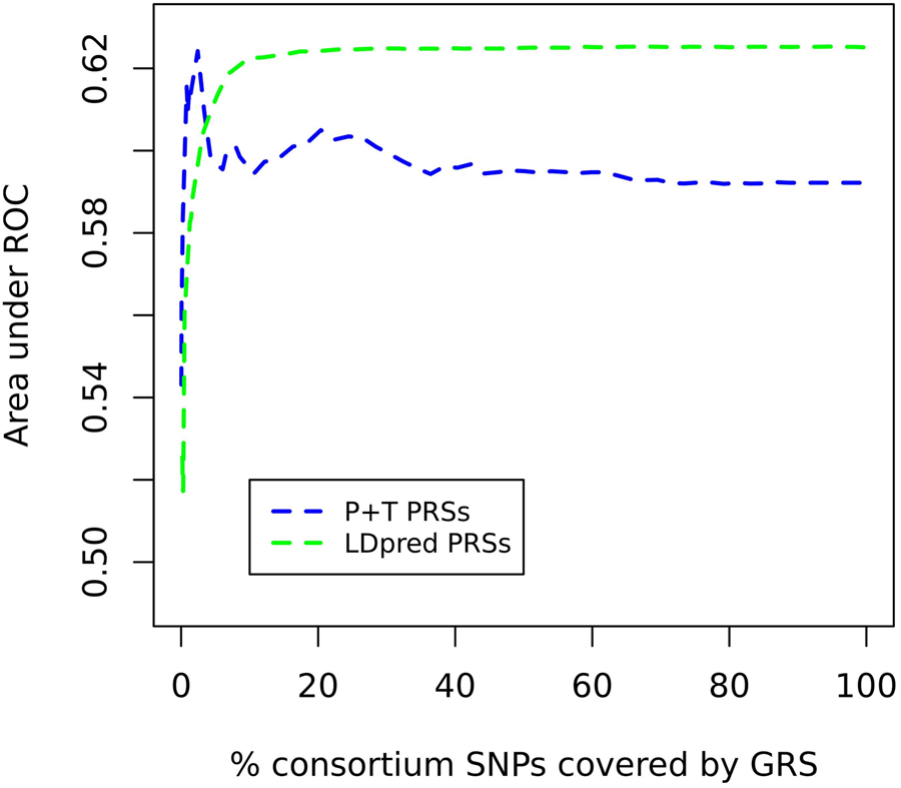
AUCs of P + T and LDpred PRSs in START. Results are for Mahajan-based PRSs derived using SNPs tested in ≥85% of the study’s maximum N_samples_ and LD from 1000 Genomes. Abbreviations: AUC, Area under the curve; PRS, Polygenic risk score; LD, Linkage disequilibrium; P + T, Pruning and thresholding; START, South Asian birth cohort; ROC, Receiver operating characteristic.

When comparing the best PRSs derivded from each method, no significant difference was observed between GraBLD, LDpred and P + T (AUCs = 0.62, Table 2, P_pairwise differences_ = 0.95). When comparing P + T to LDpred only, AUCs were higher and more stable in LDpred PRSs at P-value thresholds > 0.1 (Fig. 3).

#### Top PRS

Detailed characteristics and rankings of the best PRSs for each consortium data and each method used are shown in Supplementary Table 2. With an AUC of 0.62, the overall best (top) PRS identified in our study included 1,290,525 SNPs and was derived using the LDpred method; weights from Mahajan *et al*.; LD from 1000 Genomes; and SNPs tested in at least 93,681 samples (≥85% of the Mahajan’s maximum N_sample_).

### Association with GDM

The association results of the top PRSs with GDM (univariate models) are shown in Table 2 (continuous PRSs) and Table 3 (categorical PRSs). The odds of developing GDM was 2 to 2.5 fold higher in participants with the highest PRSs (top 25%) compared to the rest (75%) of the study population, depending on the type of PRS used. When analyzing participants with high and low PRSs values only, our results show that participants with the highest PRS values (top 25%) had between 3 and 3.4 fold increase in their risk of GDM compared to the participants with the lowest PRS values (bottom 25%). These results were similar in South Asian women from UK Biobank (Tables 2 and 3).

**Table 3:** Association results of best PRSs (categories) with GDM in South Asian women from the START and UK Biobank.

High PRS definition	Reference group	PRS type	South Asian Women					
			START			UK Biobank		
			OR	95% CI	P value	OR	95% CI	P value
Top 25%	Remaining 75%	GraBLD	2.51	1.82–3.47	1.75 × 10^−8^	2.66	1.51–4.63	0.0006
		P + T	2.08	1.51–2.87	7.44 × 10^−6^	1.80	0.99–3.17	0.05
		LDpred	2.00	1.45–2.76	2.11 × 10^−5^	2.61	1–16–3.60	0.01
Top 25%	Lowest 25%	GraBLD	3.40	2.25–5.17	7.30 × 10^−9^	5.30	2.17–15.88	0.0008
		P + T	3.09	2.10–4.74	1.47 × 10^−7^	4.21	1.67–12.82	0.005
		LDpred	3.06	2.02–4.69	1.77 × 10^−7^	3.59	1.53–9.84	0.006

Abbreviations: CI, Confidence interval; PRS, Polygenic risk score; GraBLD, Gradient boosted and LD adjusted; OR, Odds ratio; P + T, Pruning and thresholding; START, South Asian birth cohort.

## Discussion

In this study, we derived several thousands of GDM PRSs using genome-wide genotypes, large consortium data, and  different methods for use in a South Asian birth cohort. Our best PRS was built using the LDpred method, with weights extracted from the multi-ethnic analysis by Mahajan *et al*. and LD calculated using 1000 Genomes genotypes. This PRS was significantly associated with GDM in South Asian women from the START study, an observation that was successfully replicated in South Asian women from UK Biobank. Participants with the highest PRS values had an increased risk of GDM when compared to the other groups.

We observed a considerable difference in the proportion of participants with GDM between South Asian women from the START study (36.2%) and South Asian women from UK Biobank (2.2%). This disparity is likely due to major differences in the study design, recruitment strategies, and definitions of GDM between the two studies involved. For instance, the definition of GDM status in START was based on glucose levels measurements performed during pregnancy in response to an oral glucose challenge. On the other hand, GDM status was retrospectively self-reported by UK Biobank participants, which most likely resulted in some misclassification, and a reduced number of GDM cases. In an effort to refine the phenotype in UK Biobank, our control group was restricted to women without GDM who also had at least one live birth. Nevertheless, the retrospective self-reported GDM phenotype in the UK Biobank is a limitation.

Summary statistics from two large T2D GWAMAs were used to build our PRSs. One of the major advantages in using data from Mahajan *et al*. was that ~20% of its participants in their publically available data originated from the South Asian sub-continent. Although this GWAMA also included participants from other ethnic groups, the direction of association for the same reference alleles were largely similar between the South Asian and multi-ethnic samples (concordance of 70% and 92% for all variants, and nominally significant SNPs respectively, data not shown)^9^, which substantiates the use of this dataset. Mahajan *et al*.'s study also had a large maximum number of cases and controls, but many of the SNPs included in the meta-analysis were tested in a much smaller sample (Supplementary Fig. 2, Supplementary Table 1). On the other hand, no South Asian participants were included in the GWAMA performed by Scott *et al*. but the average number of samples tested for each SNP was larger than in Mahajan *et al*. Our results show that Mahajan-based PRSs consistently outperformed their Scott-based counterparts in spite of a lower genome coverage and smaller average number of participants per SNP. This highlights the importance of using consortium data of the same ethnic group than the study at hand whenever possible. However, since Mahajan *et al*.’s summary statistics were derived from a blend of participants of different ethnicities, our top PRS could likely be improved if built based on summary statistics derived from an equally powered GWAMA performed in South Asians only.

Several reports suggest that T2D and GDM share a common genetic background. In the absence of publicly available data of large GDM GWASs, summary statistics from a T2D consortium were used to derive our scores. Our results show that a T2D PRSs can be used in order to improve the prediction of GDM in South Asian women, hence confirming the hypothesis of a common genetic background between these two diseases. Assuming a good gene transferability between T2D and GDM, and a 20% of variance explained by our top P + T PRS’s SNPs, our study is well powered to detect a significant association between the PRS and GDM at a nominal level (Supplementary Table 4). Since T2D’s SNP-based heritability has recently been estimated at 0.54 (s.d. = 0.07)^56^, and given the strong significance of our top models, such assumptions seem reasonable. However, the effect size of the genetic variants could be different between the two conditions (T2D *vs*. GDM), and some loci could be specific to each disease. Although these differences should not affect our models comparisons, we expect that the predictive value of GDM PRSs will be further improved if built using weights from large GDM GWASs or GWAMAs.

Given that our methods comparison results are data driven, some of our observations only apply to cases of very similar context (e.g., use of Mahajan *et al*.), while others might be extend to a wider range of situations: Firstly, a significant conclusion derived from this study is that, whatever the consortium or the method used, restricting the list of SNPs to GWAS significant variants (P value ≤ 5 × 10^−8^) drastically reduces the predictive value of the PRSs. Unfortunately, many studies still rely on this threshold to select their loci of interest and derive their risk scores. We recommend the use of higher P-value thresholds (>0.01 in our case) whenever possible in order to increase the predictive value of the PRSs. Secondly, when comparing the best PRSs, our results suggest that the GraBLD, P + T and LDpred methods perform equally well in terms of disease prediction as measured by the AUC. Nevertheless, the identification of the optimal P + T, and LDpred PRSs required the test of several thousand predictors (n = 2,560 and 1280 respectively), when a similar result was achieved by testing 40 GraBLD models only. On the other hand, the high stability of LDpred’s AUCs when keeping SNPs with a high P-value may lead one to slightly favor the use of this method. We still recommend the use of P + T as a method of choice in cases of small number of SNPs (or low genome coverage) and reduced computational resources.

Although the discriminative capacity of the top PRS described in this analysis (AUC 0.62–0.65) and its associated risk (OR > 2) are considered as high in a context of complex traits, such values remain relatively low when compared to the predictive values of genetic variants associated with severe Mendelian disorders. In a clinical setting, such predictors remain insufficient to accurately predict future GDM, and should therefore be combined with other known GDM risk factors including age, diet or parity in order to increase the accuracy of the prediction of future cases.

In conclusion, our results show that use of predictive value of polygenic risk scores for GDM in South Asian women can be greatly improved by combining genome-wide genotyping data, extracting summary statistics from large multi-ethnic genome-wide meta-analysis and by testing and fine-tuning different parameters.

## Methods

### Study design and participants

The South Asian Birth Cohort (START): START is a prospective cohort designed to evaluate the environmental and genetic determinants of cardiometabolic traits of South Asian pregnant women and their offspring living in Ontario, Canada. The rationale and study design are described elsewhere^57^. In brief, 1,012 South Asian (people who originate from the Indian subcontinent) pregnant women, between the ages of 18 and 40 years old, were recruited during their second trimester of pregnancy from the Peel Region (Ontario, Canada) through physician referrals between July 11, 2011 and Nov. 10, 2015. All START participants signed an informed consent including genetic consent, the study was approved by local ethics committees (Hamilton Integrated Research Ethics Bard, William Osler Health System, and Trillium Health Partners), and all research was performed in accordance with the guidelines. A detailed description of the maternal measurements has been published previously^58^.

#### UK Biobank

The UK Biobank is a large population-based study which includes over 500,000 participants living in the United Kingdom^55^. Men and Women aged 40–69 years were recruited between 2006 and 2010 and extensive phenotypic and genotypic data about the participants was collected, including ethnicity and history of GDM. Details of this study are available online (https://www.ukbiobank.ac.uk)^55^. Data of South Asian women from UK Biobank were used in order to validate the results from the START study.

### Derived variables

#### START

GDM status was determined using the South Asian specific cutoffs as defined in the Born in Bradford study (fasting glucose level of 5.2 mmol/L or higher, or a 2-hour post load level of 7.2 mmol/L or higher)^4^. Self-reported GDM status was used if these measures were unavailable. Participants with a history of T2D prior to pregnancy were excluded. Using these criteria, 832 START participants with known GDM status (301 cases and 531 controls) and available genotypes were included in the analysis. The South Asian ethnicity/ancestry of participants was validated using genetic data.

#### UK Biobank

Participants in the UK Biobank completed questionnaires at several time points (questionnaire of initial assessment visit, 2006–2010; questionnaire of first repeat assessment visit, 2012–2013; questionnaire of imaging visit, 2014 onwards). For the purpose of our study, GDM cases were defined as women who self-reported having had diabetes only during their pregnancies at any time point of the study. The control group was comprised of women who: 1) had at least one child (self-reported, live births only), and 2) had never been diagnosed with diabetes or GDM in all assessments. The South Asian ethnicity/ancestry of participants was validated using genetic data.

### Consortium data

Summary statistics of the GWAS meta-analysis performed by Mahajan *et al*.^9^ and Scott *et al*.^10^ were downloaded from DIAGRAM’s main website (http://www.diagram-consortium.org).

### DNA extraction, genotyping, imputation, filtering and SNP extraction

#### Start

DNA was extracted and genotyped from a total of 867 samples (START mothers) using the Illumina Human CoreExome-24 and Infinium CoreExome-24 arrays (Illumina, San-Digeo, CA, USA). Data was cleaned using standard quality control (QC) procedures^59^ and 837 women samples passed the QC. Genotypes were subsequently phased using SHAPEIT v2.12^60^, and imputed with the IMPUTE v2.3.2 software^61^, using the 1000 Genomes (phase 3) data as a reference panel^62^. Variants with an info score ≥0.7 were kept for analysis. Addition data manipulation and SNP selection criteria for the building of the PRSs are detailed in Supplementary Information and Supplementary Fig. 1.

#### UK Biobank

A total of ~500,000 participants from the UK biobank were genotyped using the UK BiLEVE or UK Biobank Affymetrix Axiom arrays. Detailed QC, phasing and imputation procedures have previously been described^63^. As a result, 3,169 unrelated South Asian women passed QC. Among these, 2,386 participants had available GDM status respectively, and were used to replicate our PRS results from the START study. Genotypes for >98% of SNPs included in our top START GDM PRSs were available (info score ≥0.6) and were extracted for the replication.

#### 1000 Genomes

Genotypes of 1000 Genomes participants were downloaded from the project’s data portal (http://www.internationalgenome.org), and a subset of participants was created in order to match the proportion of the ethnicities represented in each consortium study.

### PRS deriving methods

#### Pruning and thresholding (P + T)

Weighted PRSs were built using GNU Parallel^64^ and PLINK v1.9 (https://www.cog-genomics.org/plink2)^65^. 64 different clump P-value cutoffs ranging from 5 × 10^−8^ to 1 were tested in order to identify the optimal index variant’s significance threshold. All other parameters were set to default.

#### LDpred

LDpred PRSs were derived using the LDpred software v0.9.9 (https://github.com/bvilhjal/ldpred)^53^. The fractions of causal variants assumed a prior were similar to the P-value thresholds used for the P + T PRSs. Since the number of SNPs was different between the PRSs, The LD radius was adjusted accordingly in each model using the recommended formula (N SNP/3000). All other parameters were kept on their default setting.

#### GraBLD

GraBLD PRSs were built using the GraBLD R package (https://github.com/GMELab/GraBLD)^54^. Data of all the women participating in the START study were used for the calibration. All parameters were set to default.

### Association analysis

The association of each PRS with GDM was assessed using a univariate logistic regression model, and areas under the receiver-operating characteristic (ROC) curves (AUCs, c-statistics) were compared in order to determine the PRS with the highest predictive value of GDM. Continuous PRSs were also divided into quartiles in order to compare the participants with highest PRS values to the other groups. Statistical significance of the difference between the predictive values of two PRSs was tested using the DeLong’s test for two correlated ROC curves. Analyses were performed using GNU Parallel^64^ and R v3.3^66^.

### Power analysis

The power to detect associations for our top P + T PRS using Mahajan *et al*.'s study characteristics as a training sample and assuming different values of proportion of variance explained by SNPs was estimated using the avengeme R package (https://github.com/DudbridgeLab/avengeme)^67^.

## Supplementary information

Supplementary Information.
