# Supplementary material for: Fine-tuning of Genome-Wide Polygenic Risk Scores and Prediction of Gestational Diabetes in South Asian Women
Source: Sci Rep. 2020 Jun 2;10:8941. doi: 10.1038/s41598-020-65360-y (PMC7265287; doi:10.1038/s41598-020-65360-y)
Supplement: Supplementary file 1 — Supplementary Information. [file 41598_2020_65360_MOESM1_ESM.pdf]

Fine-tuning of Genome-Wide Polygenic Risk Scores and Prediction of Gestational Diabetes in South Asian Women.

Amel Lamri[1,2], Shihong Mao[2], Dipika Desai[2], Milan Gupta[1,3], Guillaume Paré[2,4], Sonia S. Anand[1,2,5]

1. Department of Medicine, McMaster University Hamilton, Ontario, Canada
2. Population Health Research Institute (PHRI), Hamilton, Ontario, Canada
3. Canadian Collaborative Research Network (CCRN), Brampton, ON, Canada
4. Department of Pathology and Molecular Medicine, McMaster University, Hamilton, Ontario, Canada
5. Department of Health Research Methods, Evidence, and Impact, McMaster University, Hamilton, Ontario, Canada

Supplementary Information

Scientific Reports

2020

Data manipulation and SNP selection:

START Study: Coordinates of SNPs from Mahajan *et al.* were converted from the human genome assembly hg18 (Mar. 2006) to hg19 (Feb. 2009) using The University of California Santa Cruz (UCSC) LifOver tool (https://genome.ucsc.edu/cgi-bin/hgLiftOver). Consortium variants that met the following criteria were kept if there was the presence of: 1) valid hg19 genomic coordinates; 2) a MAF ≥0.01 in START; 3) available genotypes in START and 1000 Genomes datasets; and 4) if they had been tested in South Asian samples (for the PRSs based on Mahajan *et al.* only).

Additional PRSs were created and for which only SNPs tested in ≥ 85, 90, 95% and 95% of samples (maximum sample size in the consortium) were kept. A detailed diagram of SNP selection process for each PRS is shown in Supplementary Figure 4.

	**Mahajan *et al.*, 2014**		**Scott *et al.*, 2017**	
	Minimum $N_{samples}$ tested per SNP	N_{SNPs} remaining after filtering	Minimum N samples tested per SNP	N SNPs remaining after filtering
Keep All SNPs (ref)	25	2,324,032	4,731	6,813,331
SNPs tested in ≥85% of samples	93,681	1,602,182	134,452	6,617,007
SNPs tested in ≥90% of samples	99,192	1,305,771	142,362	6,483,925
SNPs tested in ≥95% of samples	104,703	346,290	150,275	6,146,154
SNPs tested in ≥98% of samples	108,010	223,912	155,174	5,301,848

Supplementary Table 1: Minimum sample size and number of SNPs tested in the PRSs derived from Mahajan et *al.* and Scott et *al.*

Abbreviations: ref, reference; SNP, Single nucleotide polymorphism.

Method	Consortium	LD source	Min % participants	P-value threshold	N Variants included in the PRS	% SNPs covered by PRS	Rank (within method)	Rank (all PRSs)
P+T	Mahajan *et al.*, 2014	1KG	85%	0.016	9,274	2.42	1	106
		START	95%	0.200	35,274	22.34	3	307
	Scott *et al.*, 2017	1KG	95%	0.071	73,130	9.88	211	1,077
		START	all SNPs	0.048	67,693	7.07	278	1,237
GraBLD	Mahajan *et al.*, 2014	1KG	90%	NA	1,305,596	NA	65	107
		START	90%	NA	1,305,596	NA	1	18
	Scott *et al.*, 2017	1KG	98%	NA	5,302,459	NA	961	1,894
		START	98%	NA	5,302,459	NA	641	1,458
LDpred	Mahajan *et al.*, 2014	1KG	85%	0.94	1,290,525	94.64	1	1
		START	90%	1	1,305,596	100	47	241
	Scott *et al.*, 2017	1KG	95%	0.01	112,875	2.25	426	1,800
		START	95%	0.007	88,472	1.70	834	3,199

Supplementary Table 2: Characteristics of the best PRSs by method, consortium data, and LD source.

Abbreviations: AUC, Area under the curve; PRS, Polygenic risk score; GraBLD, Gradient boosted and LD adjusted; LD, Linkage disequilibrium; Min, minimum; NA, Non applicable; P+T, Pruning and thresholding; SNP, Single Nucleotide Polymorphism.

		START				UK Biobank			
Method	Consortium	Beta	SE	P-value	AUC	Beta	SE	P-value	AUC
P+T	Mahajan *et al.*, 2014	0.448	0.08	6.55×10^{-9}	0.62	0.512	0.14	0.0003	0.64
	Scott *et al.*, 2017	0.351	0.07	2.98×10^{-6}	0.60	0.300	0.14	0.03	0.59
GraBLD	Mahajan *et al.*, 2014	0.470	0.08	1.32×10^{-9}	0.62	0.510	0.14	0.0005	0.63
	Scott *et al.*, 2017	0.342	0.07	3.93×10^{-6}	0.59	0.387	0.14	0.006	0.61
LDpred	Mahajan *et al.*, 2014	0.470	0.08	1.32×10^{-9}	0.62	0.44	0.14	0.002	0.61
	Scott *et al.*, 2017	0.281	0.07	0.00015	0.57	0.225	0.13	0.10	0.55

Supplementary Table 3: GDM association results of the best P+T, LDpred, and GraBLD PRSs in South Asian women from the START and UK Biobank studies (LD estimates derived from START).

Results are from univariate association tests with GDM. Abbreviations: AUC, Area under the curve; GraBLD, Gradient boosted and LD adjusted; NA, Non applicable; P+T, Pruning and thresholding; PRS, Polygenic risk score; SE, Standard Error; START, South Asian birth cohort.

**Proportion of variance explained by PRS SNPs**	**Power**
10%	61%
12.5%	82%
15%	94%
17.5%	99%
20%	100%
54% *	100%

Supplementary Table 4: Statistical power of the top P+T PRS assuming different portions of variances explained by the PRS's SNPs.

*T2D's SNP-based heritability as estimated by Speed, D. *et al.* Reevaluation of SNP heritability in complex human traits. *Nat Genet* **49**, 986-992, doi:10.1038/ng.3865 (2017).

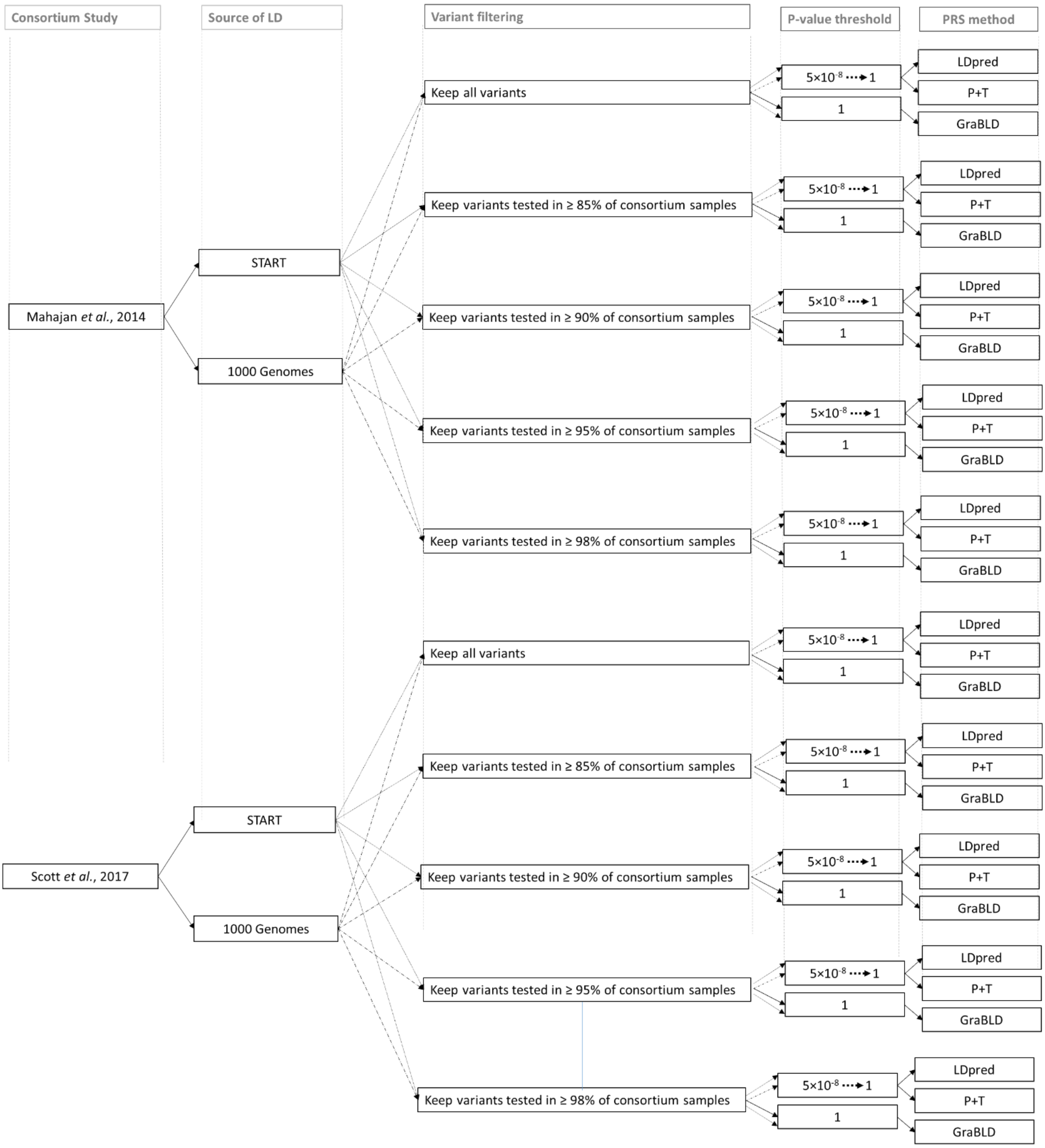

Supplementary Figure 1: Diagram of the different T2D P+T, LDpred, and GraBLD PRSs derived for South Asian women from the START study.

Abbreviations: START, South Asian birth cohort.

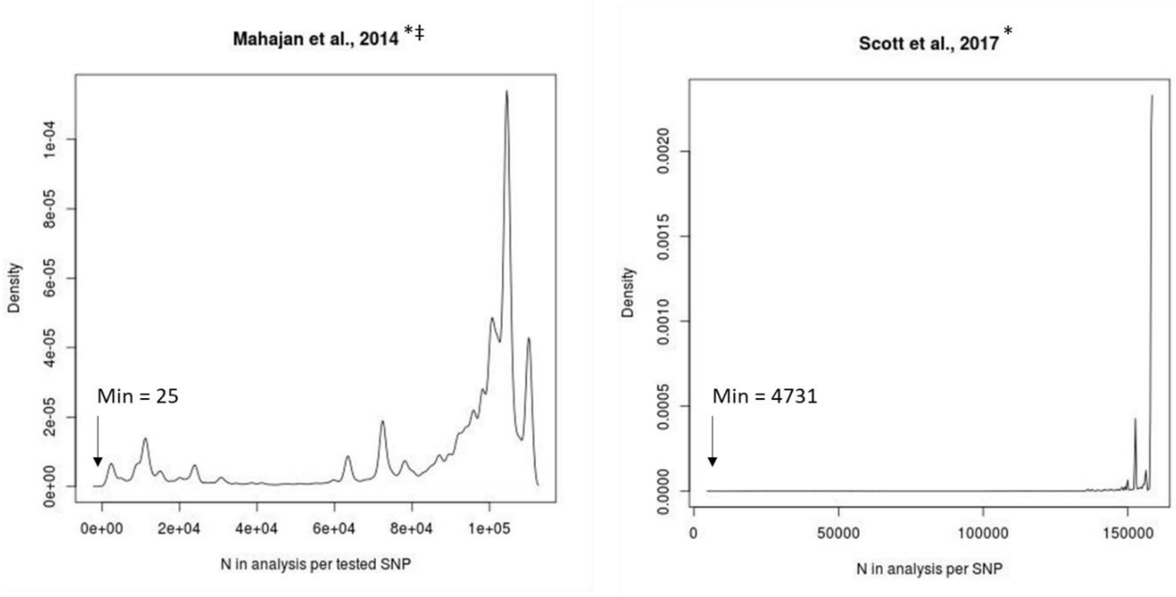

Supplementary Figure 2: Density plot of the number of participants tested for association with T2D per SNP in Mahajan *et al.* and Scott *et al.*

* SNPs common between START and the consortium data. ‡, SNPs tested in South Asians in Consortium. Abbreviations: START, South Asian birth cohort; T2D, Type 2 diabetes.

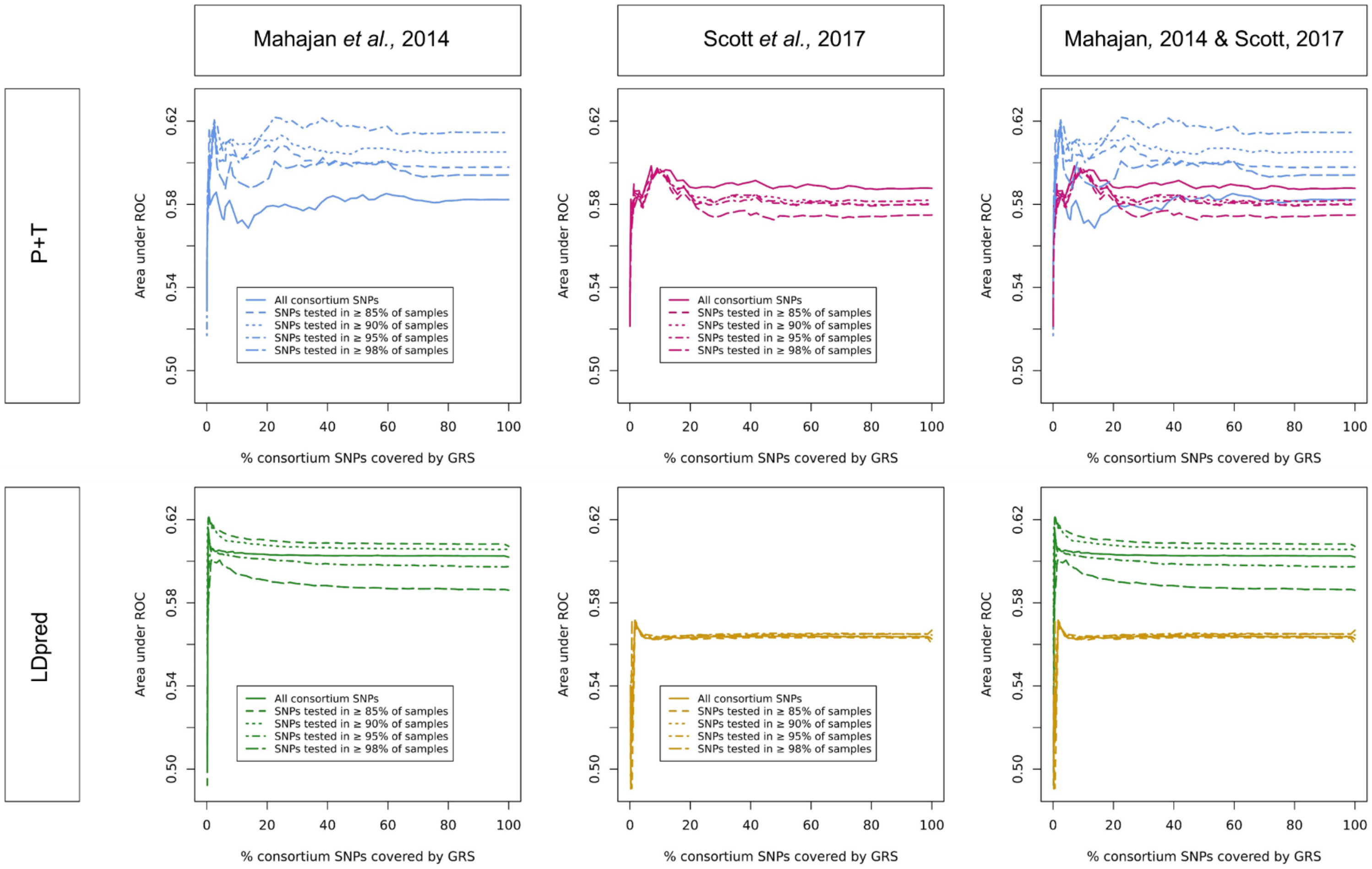

Supplementary Figure 3: AUCs of the different P+T and LDpred PRSs based on Mahajan *et al*. and Scott *et al*. (LD from START).

Results from association tests with GDM. AUC, Area under the curve; GDM, Gestational diabetes mellitus; LD, Linkage disequilibrium; P+T, Pruning and thresholding; PRS, Polygenic risk score; SNP, Single nucleotide polymorphism; START, South Asian Birth Cohort; ROC, receiver operating characteristic.

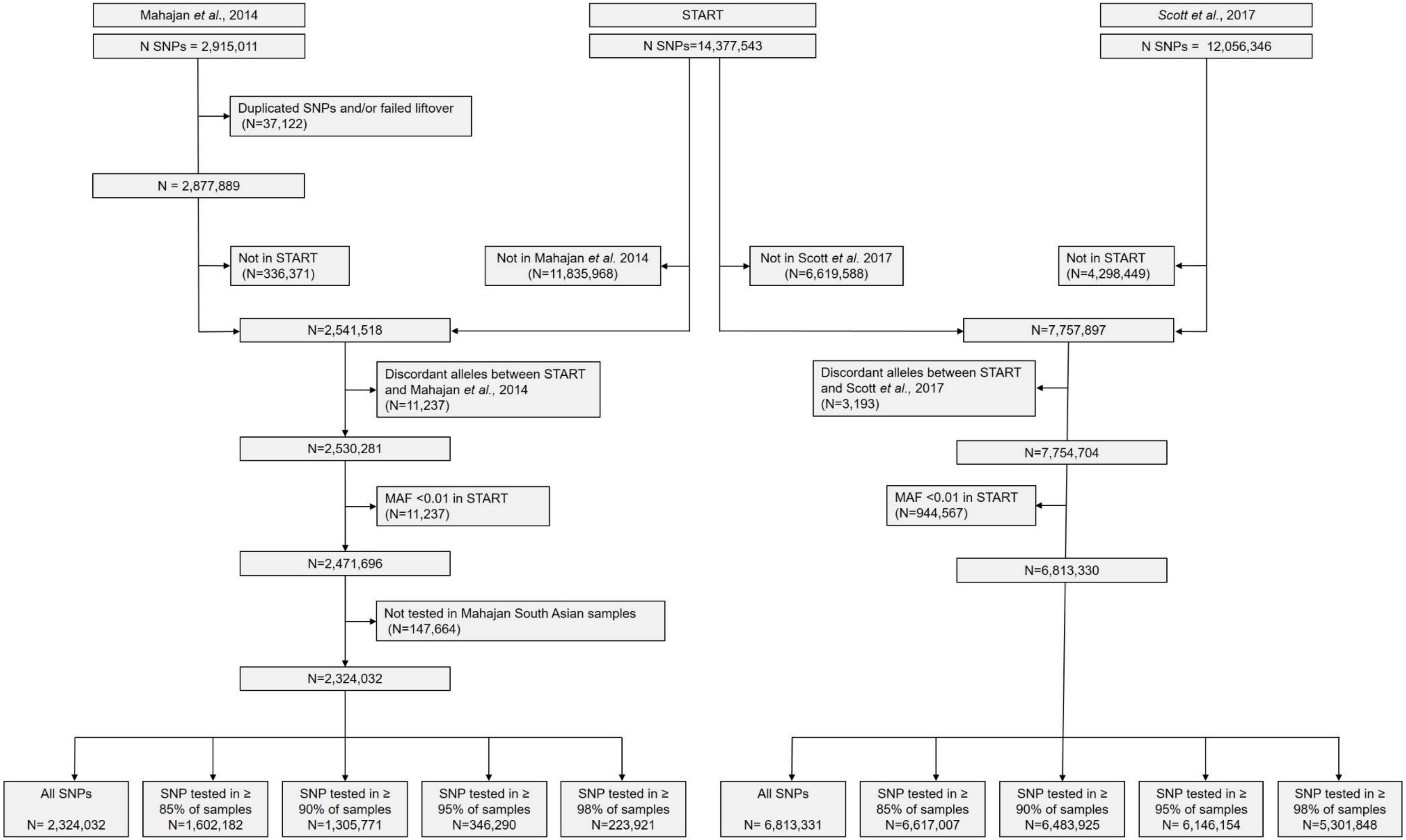

Supplementary Figure 4: Diagram of SNPs filtering steps prior to building the PRSs in START.

Abbreviations: MAF, minor allele frequency; SNP, Single nucleotide polymorphism; START, South Asian birth cohort.
